# Zinc Hydroxystannate/Carbon Nanotube Hybrids as Flame Retardant and Smoke Suppressant for Epoxy Resins

**DOI:** 10.3390/molecules28196820

**Published:** 2023-09-27

**Authors:** Congling Shi, Mei Wan, Xiaodong Qian, Jingyun Jing, Keqing Zhou

**Affiliations:** 1 Beijing Key Laboratory of Metro Fire and Passenger Transportation Safety, China Academy of Safety Science and Technology, Beijing 100012, China; 2 Faculty of Engineering, China University of Geosciences (Wuhan), Wuhan 430074, China

**Keywords:** additives, flame retardance, high performance polymers, modification, nanocomposites

## Abstract

Novel hybrid flame retardants containing zinc hydroxystannate and carbon nanotubes (ZHS-CNTs) were synthesized using the coprecipitation method, and the structure and morphology of ZHS−CNTs were investigate using an X-ray powder diffractometer (XRD), scanning electron microscope (SEM), transmission electron microscopy (TEM) and thermogravimetric analyzer (TGA). Then, the ZHS, CNTs and ZHS−CNTs were incorporated into EP, respectively, and the flame-retardant and smoke inhibition performance of the composites were compared and studied. Among the three composites, the EP/ZHS-CNT composites have the highest improvements on the fire resistance and smoke inhibition properties. With only 2.0 wt.% ZHS-CNT hybrids, the pHRR of EP/ZHS-CNT composite materials is reduced by 34.2% compared with EP. Moreover, the release of toxic gases including CO, CO_2_ and SPR from the composites was also effectively inhibited. The mechanisms of flame retardant and smoke inhibition were investigated and the improved properties were generally ascribed to the synergistic flame-retardant effects between ZHS and CNTs, the catalyzing effect of ZHS and the stable network structure of CNTs.

## 1. Introduction

In recent years, halogen-free flame retardants have become the hot spot of industrial application because the applications of traditional halogenated flame retardants are restrained by international society due to environmental reasons [1,2,3]. Among various flame retardants, the inorganic compounds have more industrial application prospects because the polymer composites with the inorganic nanofillers usually exhibit high flame retardant and smoke suppression efficiency during the combustion process [4,5,6]. Thus, various research has been carried out to fabricate polymer/inorganic nano-filler composites with improved fire safety properties [7]. Among those inorganic nano-filler composites, nano-fillers such as magnesium hydroxide (MH), zinc stannate (ZS), molybdenum (IV) sulfide (MoS_2_), zinc hydroxystannate (ZHS), carbon nanotubes (CNTs) or graphene (rGO) are introduced into the polymer matrix and the property improvements of the composites materials are investigated [8,9,10,11,12,13].

Among those nano-fillers, zinc hydroxystannate (ZHS) has various application in place of antimony trioxide as a flame retardant in the polymer materials, and it is a good candidate as an environmentally friendly smoke suppressant [14]. Generally, the Zn ion plays the effect of the Lewis acid, catalyzing the esterification and dehydrogenation of polymer materials, resulting in compact and condense char layers. Similar to the problem of other nanocomposites, some problems could not be ignored, such as the poor dispersion of ZHS due to the Van der Waals force, which will result in reduced flame retardant efficiency of ZHS [15,16]. Based on the above reasons, loading ZHS on the surface of one-dimensional or two-dimensional nanomaterials through self-assembly processes can not only solve the agglomeration problem of ZHS, but also improve the flame retardant properties efficiently due to the synergistic effects between the ZHS and nanomaterials.

As one-dimensional carbon materials, carbon nanotubes (CNTs) have been used in various fields owing to the outstanding thermal, electrical, and mechanical performances. One important application of CNTs is their use as a nano additive in polymer materials, where they can greatly improve the thermal, electrical, mechanical and flame retardant properties at low CNT loadings. Moreover, it is found that the CNTs usually absorb the radicals during thermal decomposition to interrupt the chain reaction and form the network-structured protective char layer, whose char layers can play a role in isolation and protection from thermal radiation [17,18]. Similar to ZHS and other inorganic materials, CNTs also have the poor dispersion problems in polymeric materials. Up to now, numerous modification methods have been invented to enhance the CNTs’ dispersibility, such as ultrasonication or chemical modification. Generally, the main challenges for improving the dispersity of CNTs in the polymer is how to repress the Van der Waals force among CNTs, which can be improved by physical or chemical modification.

The nanocomposites, which contain two or three nanofillers, have attracted more and more attention due to the synergy in flame retardancy [19,20]. As for CNTs, the network structures of CNTs in the polymer matrix can contribute to the improvement of mechanical, electrical, flame resistant and thermal performances of the composite materials, but the aggregation effects of CNTs in the polymer limit its further application. Moreover, ZHS is not only a good smoke suppressant, but also a good flame retardant synergist. It is reported that ZHS can form a synergistic flame retardant system with the other flame retardants such as ammonium polyphosphate (APP). Therefore, a combination of ZHS and CNTs has synergistic flame retardant potential, which can improve the flame retardancy and smoke suppression efficiency of the nano-filler in the polymer matrix [21,22].

Epoxy resins (EP) are widely used as a matrix in composite materials due to their excellent adhesion, mechanical properties, insulation and chemical corrosion resistance, resulting in good impregnation and fiber adhesion [23]. In fields such as the aviation industry, the use of EP composite materials has been increasing, but the flammability of EP has always limited its application [24]. Therefore, reducing the flammability of EP has become the key to improve the application of polymer composite materials. In addition, EPs release a large amount of smoke particles during the combustion processes, making it particularly important to improve the flame retardancy and smoke suppression performance of flame retardants [25].

In this manuscript, novel hybrid flame retardants containing zinc hydroxystannate and carbon nanotubes (ZHS-CNTs) were synthesized using the coprecipitation method. The ZHS−CNTs were introduced into the EP matrix, and the fire safety of the EP/ZHS−CNTs composites were compared and studied, the flame-retardant mechanism was also discussed. The flame retardance and smoke suppression performances have prominently improved due to the synergic flame retarding effects between ZHS and CNTs, the catalyzing effect of ZHS and the stable network structures of CNTs.

## 2. Discussion

### 2.1. Characterization of ZHS and ZHS−CNTs Hybrids Flame Retardants

**Figure 1:**
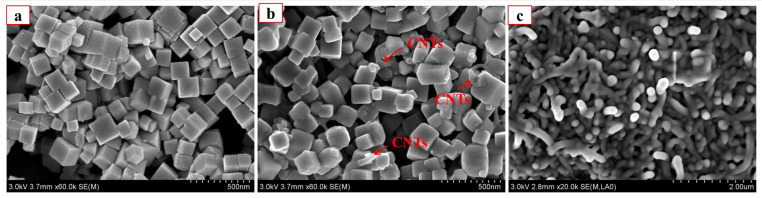
(**a**) The XRD result of ZHS, ZHS−CNTs and CNTs; SEM images of ZHS (**b**), ZHS−CNTs (**c**) and CNTs.

The X-ray diffraction pattern (XRD) of ZHS, ZHS−CNTs and CNTs are shown in Figure 1a. As for the XRD of CNTs, the two peaks at the location of 25.43° (002) and 42.4° (100) correspond to the hexagonal graphite structures. As for ZHS, it is found that the main diffraction peaks are in accordance with the primitive standard cubic phase ZnSn(OH)_6_ (JCPDS: NO. 74-1825), indicating that the purity of the ZHS is high [14,26]. The XRD pattern of ZHS−CNTs hybrids flame retardants is in accordance with ZHS, indicating that the fabrication processes of ZHS−CNTs have no influence on the crystalline structure of ZHS. From the diffraction pattern of ZHS−CNTs hybrids, the intensity of CNTs’ peaks decreased significantly. This indicates that the presence of ZHS in the ZHS−CNTs hybrids has an impact on the interaction among CNTs.

**Figure 2:**
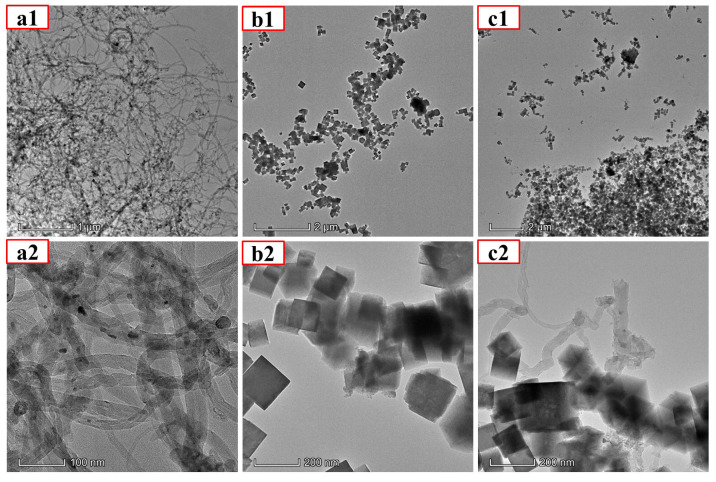
TEM images of CNTs ((**a1**) ×200, ((**a2**) ×2000), ZHS ((**b1**) ×100, ((**b2**) ×1000) and ZHS−CNTs ((**c1**) ×100, ((**c2**) ×1000).

**Figure 3:**
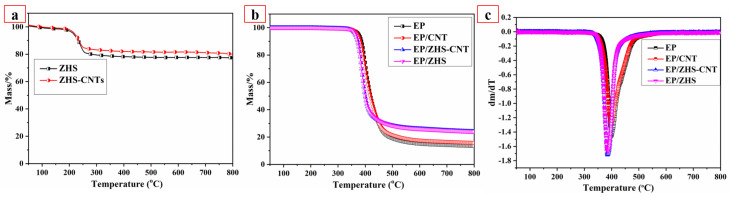
TGA profiles for ZHS and ZHS−CNTs under nitrogen atmospheres (**a**); TGA (**b**) and DTG (**c**) profiles for EP and its composites as function of temperature under nitrogen atmosphere.

Figure 1 shows the SEM images of ZHS, CNTs, and ZHS−CNTs hybrids. As for ZHS, it exhibits a cube structure, and the side length of ZHS is about 200 nm. As for the ZHS−CNTs hybrid flame retardants, ZHS and CNTs appear on the same screen, and CNTs disperse on the surface or ran through the ZHS cube, confirming the formation of the ZHS−CNTs hybrid flame retardants instead of a physical mixture of the two components. As shown in Figure 2, the microstructure morphology of ZHS, CNTs and ZHS−CNTs compounds are also characterized by TEM. It can be found that the diameter of CNTs is around 9 nm, and as for ZHS, it is obvious that ZHS has a square structure and the large surface area of ZHS is conducive to playing its catalytic role in reducing the fire risk. The structure of the ZHS−CNTs hybrid flame retardant is shown in Figure 2, and CNTs are well attached to or cross through ZHS. Because the functional groups such as -COOH of CNTs and Zn have interaction effects, the CNTs can serve as the support for ZHS in the square structure. As for the TEM of ZHS−CNTs hybrids at high resolution, it can also be found that the CNTs crossed or adsorbed on the surface of ZHS. It can be concluded from the above the XRD and SEM results that ZHS and CNTs have been perfectly combined in nano-scale as one flame retardant hybrid. The CNTs can act as the barrier and are well attached to or cross through ZHS, which can weaken the Van der Waals force among CNTs. The interaction between CNTs and ZHS will be conducive to promote the dispersion of ZHS and CNTs in the polymer. TGA results of ZHS and ZHS−CNTs hybrids are shown in Figure 3a. Both ZHS and ZHS−CNTs hybrids show the main mass loss at 200–300 °C in nitrogen atmosphere. Compared with ZHS, the char residues of ZHS−CNTs hybrid flame retardants increase slightly at 800 °C, which is mainly due to the synergistic effects between ZHS and CNTs. 

### 2.2. The Thermal Properties of EP and Its Composites

The TGA results are shown in Figure 3 and Table 1. It is obvious that all the composites have only a one-stage degradation process, attributing to the decomposition of the epoxy molecular chains. With the incorporation of CNTs, ZHS and ZHS-CNTs, the thermal stability of all the epoxy resin composites is enhanced significantly. As for the char residues, it can be found that they increased at high temperature. For instance, the char residues at 700 °C are only 16.5% for the EP/CNTs composites. And the char residues of EP/ZHS composites increased to 24.1%. Especially when 2% ZHS−CNTs flame retardant is added to EP, the carbon residues increased further to 24.8%, showing excellent improvements. Combined with the previous research, it can be found that the introduction of ZHS or ZHS−CNTs result in the early decomposition of EP composites at low temperature, while the T_max_ of the composite materials are decreased slightly. Taking into account the above TGA results, it can be concluded that ZHS−CNTs hybrid flame retardants can slightly decrease the T_max_ to some extent and catalyze the formation of carbon.

### 2.3. Flame Retardant Properties of EP and Its Composites

**Figure 4:**
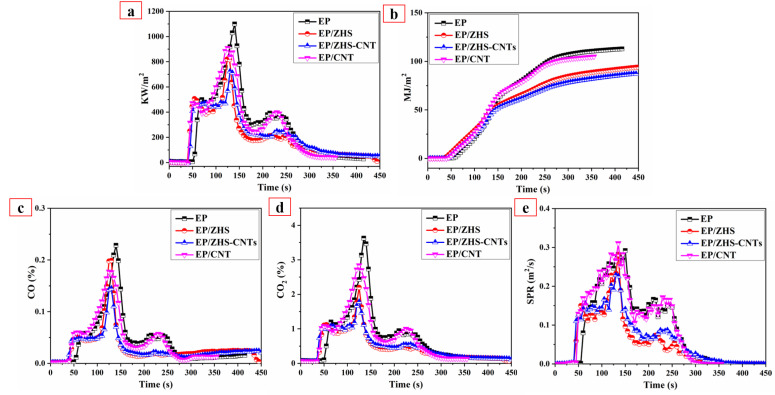
Cone calorimeter test results: HRR (**a**) and THR (**b**) curves of EP and its composites; smoke releasing of EP and its composites during the cone test, CO (**c**), CO_2_ (**d**) and SPR (**e**).

The burning of polymeric materials can not only produce a lot of heat, but also a lot of toxic gases. Thus, the releases of the heat and fumes during the combustion are very important for estimating the flame retardance of polymer materials. The fire safety property of polymeric materials is studied by the cone calorimeter, and the characteristic parameters including peak heat release rate (pHRR), total heat release (THR), smoke production rate (SPR) and CO and CO_2_ release are shown in Figure 4.

The HRR and THR curves of EP and its composites are shown in Figure 4a,b. It can be clearly observed that the pHRR value of EP is 1102 kW/m^2^. With only 2.0 wt.% ZHS−CNTs hybrids in the EP matrix, the pHRR of EP/ZHS−CNTs composites is reduced by 34.2% compared with EP; and as for those with 2.0 wt.% ZHS in the EP, the reduction is only 24.4 wt.%. Similar to the results for TGA, the pHRR of EP/ZHS−CNTs was as low as 725 kW/m^2^, with the most significant improvement. Moreover, it is observed that the THR of EP/ZHS−CNTs composite materials was reduced by 20.5% in comparison with pure EP. Due to the synergistic effect between CNTs and ZHS, the flame retardancy of EP/ZHS−CNTs composites is remarkably promoted. Generally speaking, the combination of CNTs and ZHS at the nanoscale can inhibit the polymerization of carbon nanotubes and ZHS, catalyze the formation of carbon, and form the continuous and dense char layer as a physical barrier to effectively prevent the diffusion of combustible gas and heat transfer, so as to prevent the further combustion of epoxy composites [17].

Toxic smoke is very harmful to human health and is considered to be the main cause of death in fire accidents. The CO and CO_2_ release and SPR results of the composites during the cone test are shown in Figure 4. It is apparent that the peak CO and CO_2_ releasing values of all the flame-retardant composite materials decreased, and the values of the EP/ZHS−CNTs composites are the lowest among all the composites. As for the smoke releasing, the ZHS−CNTs hybrids also have a good effect on the reduction of SPR. According to the carbon balance theory, the ZHS−CNTs hybrids can reduce the release of carbon-based gases, and they can also catalyze the char formation as correspondence. The improved performance in the smoke suppression properties is beneficial for personnel safety in fire accident.

### 2.4. The Char Layers of EP and Its Composites

**Figure 5:**
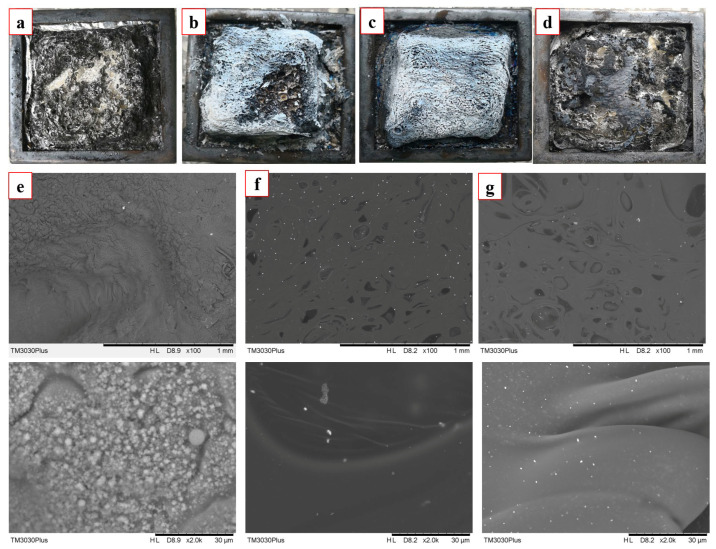
Digital photos of the char residues after the cone test: (**a**) EP; (**b**) EP/ZHS composites; (**c**) EP/ZHS−CNTs composites; (**d**) EP/CNTs composites; SEM of the char residues of (**e**) EP/CNTs, (**f**) EP/ZHS−CNTs and (**g**) EP/ZHS after the cone test.

As we all know, the char layers have a direct relationship with the flame retardant properties during combustion, hence it is of practical significance to analyze the residual char layers after the cone test with the aim of better understanding the fire retardant mechanism [27,28]. The residual chars of the composites are shown in Figure 5a–d. As for the digital photos of the samples, the char layers of EP are very few. As for char layers of the composites, it is found that the incorporation of ZHS can catalyze the formation of char. Moreover, the char layer of ZHS-based composites appear white. As those for EP/ZHS−CNTs composites, it is obvious that they are more complete than those of EP/ZHS composites, which may be mainly owing to the catalytic carbonization impact of ZHS and the char strengthening effect of CNTs [29,30].

Figure 5e–g shows the SEM results of the carbon residues after the cone calorimeter test. As for EP, the entire char layer of EP exhibits uneven and loose morphology, which is extremely uneven. As for the EP/ZHS and EP/ZHS−CNTs composites, it is clear that the char residues are more continuous and compact, which is due to the synergistic char layer enhancement of CNTs and ZHS at high temperatures. According to previous work, CNTs can form network-structured protective layers and ZHS has a catalytic carbonization ability to improve the flame retardance of polymer materials [17]. The ZHS−CNTs hybrids in the composites result in condensed and insulating char to serve as protective char layers to protect the polymer matrix form thermal radiation.

**Figure 6:**
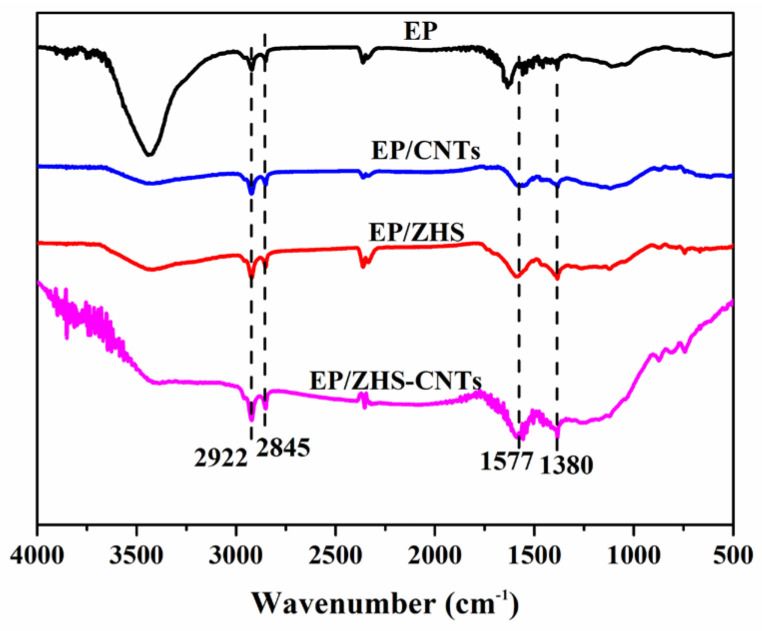
FTIR of the char layers after the cone test.

**Figure 7:**
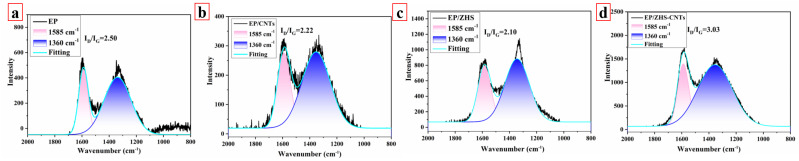
The Raman of the char layers of EP (**a**), EP/CNTs (**b**), EP/ZHS (**c**) and EP/ZHS−CNTs (**d**) after the cone test.

As shown in Figure 6, the char layers have been tested and analyzed by FTIR. Generally, the sharp peaks at 1380 cm^−1^ and 1577 cm^−1^ correspond to the vibrations of C=C in the aromatic compounds, but the peaks at 1577 cm^−1^ shifted to lower wavenumbers after the CNTs-ZHS hybrids were introduced into the EP matrix. Moreover, the peaks belonging to alkane are detected at 2922 cm^−1^ and 2845 cm^−1^ [31,32]. Meanwhile, the carbon residues have been further investigated by Raman spectroscopy. As shown in Figure 7, the two peaks at around 1360 and 1585 cm^−1^ correspond to the D band and G band. The D band and G band pertain to the disordered graphite and crystalline graphite, respectively, and the ratio of the intensity of the D and G bands (I_D_/I_G_) reflect the degree of graphitization of the char layers [33,34]. It can be observed in Figure 7 that the I_D_/I_G_ ratio was arranged in the order EP/ZHS < EP/CNTs < EP < EP/ZHS-CNTs. The I_D_/I_G_ ratio of EP/ZHS−CNTs composite was the highest, demonstrating that the ZHS−CNTs in the EP matrix can only catalyze the formation of amorphous carbon. However, the TGA results suggested that the ZHS or ZHS−CNTs hybrids can play a catalytic role in promoting the formation of char layers. Taking into account the above test results, it is apparent that the ZHS−CNTs hybrids can only catalyze the formation of more vitreous carbon during the combustion processes [35]. To sum up, the improved fire safety performances were attributed to the synergistic flame retardation between ZHS and CNTs, the catalytic effect of ZHS and the stable network structure of CNTs.

## 3. Materials and Methods

### 3.1. Raw Materials

Epoxy resins (EP) were supplied by Hefei Jiangfeng Chemical Industry Co. (E-44, epoxy value: 0.44). Zinc sulfate heptahydrate (Zn_S_O_4_·7H_2_O), 4,4-diamino-diphenyl methane (DDM) and sodium stannate trihydrate (Na_2_SnO_3_·3H_2_O) were provided by Sinopharm Chemical Reagent Co. Ltd. (Shanghai, China). Additionally, carbon nanotubes (CNTs) were provided by Chengdu Organic Chemicals Co., Ltd. (Chengdu, China).

### 3.2. Preparation of ZHS and ZHS−CNTs Hybrids Flame Retardants 

The preparation of ZHS is as follows: 600 mL of deionized water and 20 mmol of ZnSO_4_·7H_2_O were mixed in 1000 mL of three-neck flask at 25 °C. Then, the equal mole of Na_2_SnO_3_·H_2_O was dripped into the above three-neck flask, and the mixtures were stirred for another 4 h, then the white suspension was filtered and washed by deionized water, and dried overnight in a vacuum oven to obtain the desired product.

**Scheme 1:**
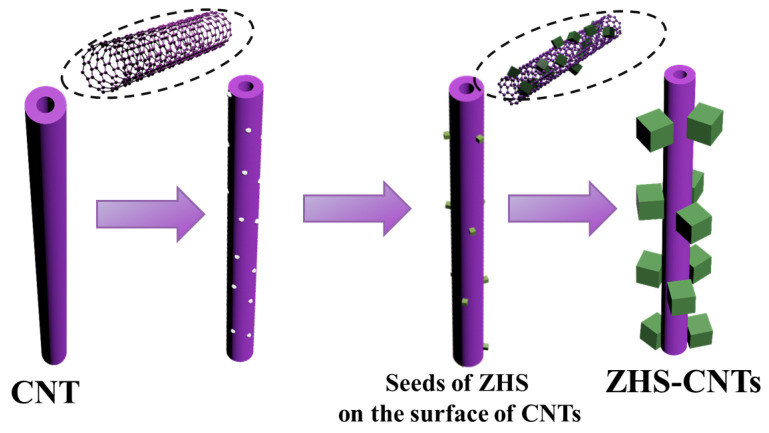
The preparation processes of ZHS−CNTs hybrid flame retardants.

The preparation of ZHS−CNTs hybrids (ZHS-CNTs) is as follows: ZnSO_4_·7H_2_O (10 mmol), CNT (0.4 g) and deionized water (300 mL) were mixed in 500 mL three-neck flask at 25 °C. Then, the equal mole of Na_2_SnO_3_·H_2_O was dripped into the above three-neck flask, and stirred for 4 h to obtain a well-mixed solution, then the ZHS−CNTs hybrid was obtained by filtering and washing the black suspension three times and vacuum drying. The yield of ZHS−CNTs was 0.51 g. The synthesis processes of ZHS−CNTs hybrid flame retardants are illustrated in Scheme 1.

### 3.3. Preparation of Flame-Retardant Epoxy Composites

Briefly, the epoxy composites with 2 wt.% ZHS−CNTs hybrids were prepared as follows: epoxy resins (100 g) and ZHS−CNTs (2.4 g) were added into a 250 mL three-necked flask. Then, the temperature of the mixtures was increased to 100 °C and continuously stirred at this temperature until ZHS−CNTs was well dispersed in the epoxy resins. And then, DDM (21.7 g, meltdown) was added into epoxy resins. Then, the mixtures were cured at 100 °C for 2 h and 150 °C for 2 h, respectively. After that, the EP/ZHS−CNTs composites were obtained. For comparison, the EP/CNTs and EP/ZHS composites with 2 wt.% flame retardants loadings were prepared by the same method. The composition of the composites is listed in Table 1.

### 3.4. Characterization

The FTIR spectroscopy of the flame retardant and char layers were studied by the Nicolet 6700 FT-IR spectrophotometer, and the wavelength ranges were from 4000 to 500 cm^−1^. 

The morphology and structures of the nano-fillers were operated on transmission electron microscopy (TEM) (JEOL JEM-2100). The flame retardants were dissolved in ethanol and dripped onto copper grids after ultrasonication. 

The thermal stability of the composites was experimentally tested with a thermogravimetric analyzer (TGA 550, TGA 550, New Castle, DE, USA) in the air or nitrogen atmosphere. In the range of 50–700 °C, 5–10 mg samples were heated at a heating rate of 20 °C/min. 

The cone calorimeter test (CCT, FTT Cone calorimeter (East Grinstead, UK)) was adopted to investigate the flame-retardant properties of the composites with the specimen size of 100 mm × 100 mm × 3 mm, and the heat flux of CCT was 35 kW/m^2^.

The scanning electron microscope (SEM) was adopted to investigate the surface structures of the composites. 

Raman spectrometer (Renishaw inVia, New Mills, UK, laser wavelength of 632.8 nm) was used to analyze the chemical structure of residual carbon after CCT. The binding energy was recorded in the range of 500–2500 eV.

## 4. Conclusions

Novel ZHS−CNTs hybrid flame retardants were prepared by in situ synthesis of ZHS and incorporated into EP resins. The structures of the hybrids were researched by XRD, SEM and TEM, confirming the successful fabrication of ZHS−CNTs hybrids. TGA results indicated that the ZHS−CNTs hybrid flame retardants in the EP matrix can improve the char residues compared with CNTs and ZHS alone. With only 2.0 wt.% ZHS−CNTs hybrids in the EP matrix, the pHRR of EP/ZHS−CNTs composites is reduced by 34.2% compared with EP. Moreover, the toxic gases released such as CO and CO_2_ and the SPR of the composites were also effectively inhibited. The flame retardant mechanism was the synergistic flame retardation between ZHS and CNTs, the catalyzing effect of ZHS and the stable network structures of CNTs. This work expands the new content of flame retardant research for EPs.

## Figures and Tables

**Table 1:** The TGA data for pure EP and its composites.

Samples	T-10%/°C	T_max_/°C	Char Residues at 700 °C/%
EP	386	399	13.9
EP/CNTs	382	397	16.5
EP/ZHS	371	384	24.1
EP/ZHS-CNTs	371	386	24.8

## Data Availability

Not applicable.

## Sample Availability

Available.
